# Structural and Mutational Analysis of *Escherichia coli* AlkB Provides Insight into Substrate Specificity and DNA Damage Searching

**DOI:** 10.1371/journal.pone.0008680

**Published:** 2010-01-13

**Authors:** Paul J. Holland, Thomas Hollis

**Affiliations:** Department of Biochemistry, Wake Forest University School of Medicine, Winston-Salem, North Carolina, United States of America; University of Minnesota, United States of America

## Abstract

**Background:**

In *Escherichia coli*, cytotoxic DNA methyl lesions on the N1 position of purines and N3 position of pyrimidines are primarily repaired by the 2-oxoglutarate (2-OG) iron(II) dependent dioxygenase, AlkB. AlkB repairs 1-methyladenine (1-meA) and 3-methylcytosine (3-meC) lesions, but it also repairs 1-methylguanine (1-meG) and 3-methylthymine (3-meT) at a much less efficient rate. How the AlkB enzyme is able to locate and identify methylated bases in ssDNA has remained an open question.

**Methodology/Principal Findings:**

We determined the crystal structures of the *E. coli* AlkB protein holoenzyme and the AlkB-ssDNA complex containing a 1-meG lesion. We coupled this to site-directed mutagenesis of amino acids in and around the active site, and tested the effects of these mutations on the ability of the protein to bind both damaged and undamaged DNA, as well as catalyze repair of a methylated substrate.

**Conclusions/Significance:**

A comparison of our substrate-bound AlkB-ssDNA complex with our unliganded holoenzyme reveals conformational changes of residues within the active site that are important for binding damaged bases. Site-directed mutagenesis of these residues reveals novel insight into their roles in DNA damage recognition and repair. Our data support a model that the AlkB protein utilizes at least two distinct conformations in searching and binding methylated bases within DNA: a “searching” mode and “repair” mode. Moreover, we are able to functionally separate these modes through mutagenesis of residues that affect one or the other binding state. Finally, our mutagenesis experiments show that amino acid D135 of AlkB participates in both substrate specificity and catalysis.

## Introduction

DNA in cells is constantly exposed to alkylating agents from both the external environment as well as internal cellular metabolic processes. These alkylating compounds react primarily with oxygen and nitrogen atoms on DNA bases [1], [2] generating lesions that if left unrepaired can be cytotoxic and mutagenic [3]. To circumvent the effects of methylation damage cells have evolved DNA repair pathways that include nucleotide excision repair, base excision repair, and direct repair pathways that specifically recognize alkylated bases and efficiently repair the lesion [1], [3].

The DNA direct repair protein, *Escherichia coli* AlkB, repairs DNA alkylation damage that primarily occurs from S_N_2 alkylating agents [4], [5]. AlkB is a member of the iron(II) dependent 2-oxoglutarate (2-OG) dioxygenase superfamily [6] that couples decarboxylation of 2-OG to succinate to oxidation of methylation damage primarily in the form of 1-methyladenine (1-meA) and 3-methylcytosine (3-meC) upon binding of dioxygen to the iron(II) cofactor [7], [8], [9]. The AlkB enzyme recognizes and repairs other alkylated DNA lesions such as 3-methylthymine (3-meT) [10], 1-methylguanine (1-meG) [11], [12], and 1,N6-ethenoadenine (εA) [13], albeit with lower efficiency. AlkB specificity is limited to the N1 position of purines and N3 of pyrimidines [14], which is in contrast to DNA glycosylases that remove lesions on the N3 position of purines and less frequently the O2 position of pyrimidines [2].

The demethylase activity of AlkB is conserved throughout prokaryotes and eukaryotes. There are at least nine AlkB genes identified in human cells, underscoring the importance of this mechanism in the maintenance of the genome. Four of these human enzymes have been shown to share a similar oxidation mechanism with the *E. coli* AlkB enzyme. The human AlkB homolog 1 (hABH1) is a mitochondrial protein that demethylates 3-meC in both RNA and DNA [15]. The hABH2 protein is specific for 1-meA damage in dsDNA [16], and hABH3 recognizes and repairs methylation damage in ssDNA as well as RNA [7]. A fourth homolog, the obesity related FTO (fat mass and obesity associated) protein, primarily recognizes 3-meT lesions [17], and is required for central nervous system and cardiovascular system development [18].

Recent crystal structures of *E. coli* AlkB:DNA complexes [19], [20], ABH2:DNA complexes [20], and hABH3 [21] have provided some initial insight into mechanisms responsible for substrate binding and catalysis of AlkB proteins. Despite the existing information regarding AlkB:DNA recognition and activity, there are still open questions regarding the substrate specificity for the AlkB protein as well as its recognition strategies for single-stranded (ssDNA) versus double-stranded DNA (dsDNA). AlkB repairs methylated DNA damage in ssDNA much more efficiently than in duplex DNA [7] and displays significantly different activity on structurally similar methylated bases. In order to further define the nucleic acid interactions, as well as understand the active site architecture that defines substrate specificity we coupled X-ray crystallography studies of the *E. coli* AlkB protein in complex with a 17 nucleotide stretch of ssDNA containing a 1-meG lesion with biochemistry analysis of *E. coli* AlkB mutants. The biochemical data and the structural data reveal new insights into DNA binding, substrate specificity, and new structural dynamics that establish a model for how AlkB searches for DNA methylation damage in ssDNA.

## Results and Discussion

### Structure of the *E. coli* AlkB/1-meG Complex

**Figure 1:**
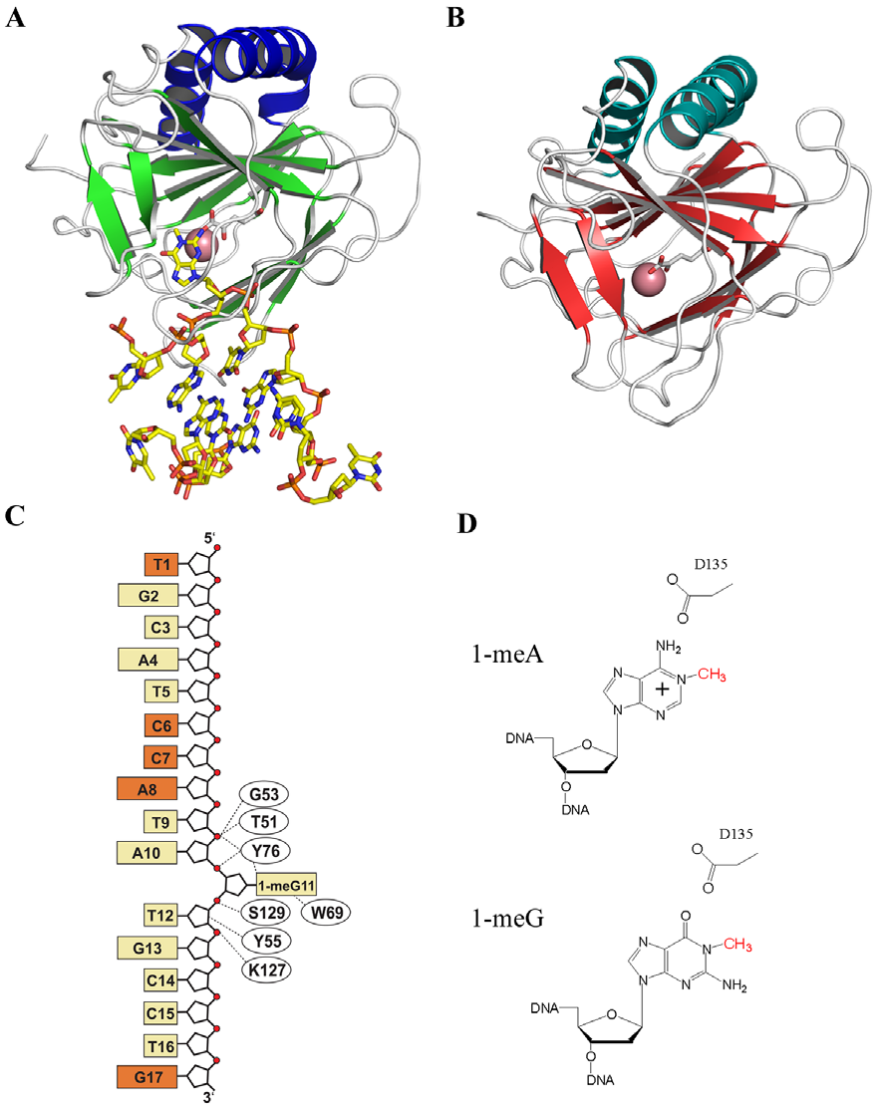
AlkB structures. (**A**) Ribbon structure of D135A *E. coli* AlkB in complex with ssDNA containing 1-meG and cofactors Co(II) and 2-oxoglutarate (2-OG). (**B**) Structure of the full length wild-type AlkB holoenzyme in complex with Co(II) and 2-OG. (**C**) Schematic of the single-stranded oligonucleotide used for crystallization. Protein residues forming direct hydrophobic or hydrogen bonding interactions with the DNA are indicated. The dark orange boxes represent the bases that were disordered and not modeled into this structure. (**D**) Schematic of 1-methyladenine and 1-methylguanine bases with the lesion in red. Residue D135 provides substrate specificity for adenine and cytosine through favorable interactions with the exocyclic amines of the bases whereas guanine and thymine bases are subject to electrostatic clashes of oxygens.

We determined the structure of the full length *E. coli* AlkB protein in complex with a 17-mer ssDNA oligonucleotide containing a 1-meG lesion to 2.2 Å resolution and the structure of the AlkB holoenzyme in the absence of nucleotide substrate to 2.9 Å resolution (Table 1 and Figure 1). The structure of the ssDNA complex was determined using a D135A mutation of AlkB because our mutagenesis data revealed that this residue is responsible for the specificity of 1-meA and 3-meC lesions. Our DNA binding experiments further showed this mutant has a higher affinity for DNA that allowed us to crystallize the DNA complex without the need for cross-linking as was done for the dsDNA complex [20]. The protein was crystallized under aerobic conditions in complex with 2-OG and cobalt(II) as the metal ion. The use of cobalt supports DNA binding but prevents catalytic activity [22]. Although less prevalent than 1-meA, 1-meG has been shown to be present *in vivo* and if left unrepaired contributes to the mutagenesis of DNA within cells [12], [23]. We determined the structure in complex with ssDNA containing a 1-meG base to understand the preference of AlkB for ssDNA substrates over dsDNA and elucidate structural features responsible for substrate specificity. The structures were solved by molecular replacement using the AlkB structure as a search model (pdbid: 2FD8) [19]. Interestingly, in the crystals of the AlkB-ssDNA complex two monomers crystallized in the asymmetric unit, but only one monomer was bound to DNA. Thus, a second nucleotide-free form of this mutant was also determined. Of the 17 nucleotides present in the ssDNA oligonucleotide, only 12 were able to be modeled into the electron density of the structure (Figure 1).

**Table 1:** Crystallographic data and statistics.

Crystal	Asp135Ala AlkB:1-meG DNA (Co/2-OG)	Wt AlkB unliganded (Co/2-OG)
**Spacegroup**	C2	C2
**Molecules/asu**	2	2
**Unit Cell Parameters (** ***a*** **, ** ***b*** **, ** ***c*** **)(Å)**	147.81×41.44×85.62	148.55×42.90×86.53
**(α, β, γ)(°)**	90, 120.98, 90	90, 121.78, 90
**Resolution range (Å)**	42.69–2.15(2.23–2.15)	30.02–2.90(2.98–2.90)
**Unique Reflections**	22909	9374
**Redundancy**	6.94 (6.27)	4.21 (4.43)
**Completeness (%)**	99.7 (99.9)	93.6 (76.74)
**R_merge_** a	0.104 (0.396)	0.113 (0.422)
**Mean I/σ**	9.4 (3.4)	6.8 (2.9)
**R_work_ (%)** b	21.1	23.0
**R_free_ (%)**	27.3	30.7
**RMSD Bond Lengths (Å)**	0.009	0.006
**RMSD Bond Angles (°)**	1.619	1.062

a *Rmerge = Σ|I−<I>|/ΣI, where I is the observed intensity and <I> is the average intensity.*

b *Rfactor = Σ||F_o_|−|F_c_||/Σ|F_o_|. Rfree is the same as R, but calculated with 5% of the reflections that were never used in crystallographic refinement.*

*Values in parentheses are for highest resolution shells.*

**Figure 2:**
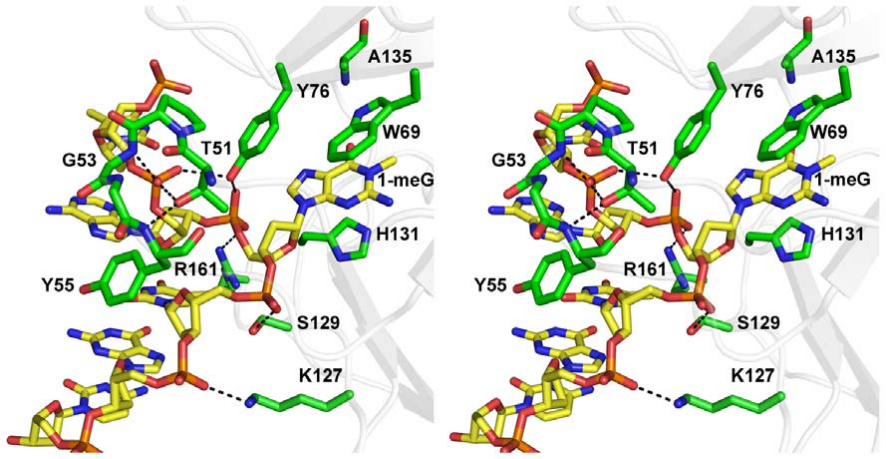
The AlkB-ssDNA structure (stereo image) shows amino acids important for ssDNA binding. Residue D135 provides substrate specificity for adenine and cytosine through an electrostatic interaction with the exocyclic amines of the bases. Stacking of the methylated base in the active site against residue W69 is required for catalysis. Residue T51 is in a loop that makes several contacts to the DNA backbone and seems to participate in the initial binding of the protein to DNA to search for methylated bases.

The protein utilizes both hydrophobic interactions and hydrogen bonding that stretch across five nucleotides to maintain the protein-DNA complex (Figure 1). The phosphodiester backbone of the ssDNA binds in an electropositive DNA binding groove that is created by a T51-Y55 DNA binding loop, S129, and K127 (Figure 1, 2). The interactions of the binding groove provide the stability to maintain the flipped conformation of 1-meG in the substrate binding pocket. The substrate base is bound in the active site pocket formed by hydrophobic stacking interaction of W69 and H131. The methyl group of the 1-meG is adjacent to the bound metal ion and properly positioned for oxidative repair. The hydroxyl group of T51 and backbone amide of G53 form direct hydrogen bonding interactions to the phosphate of the nucleotide 5′-adjacent the 1-meG base (Figure 2). Amino acids Y76, K127 and S129 provide additional contacts with the phosphodiester backbone, while the side chain of Y55 forms a hydrophobic packing interaction with the ribose sugar. The general interactions between the protein and phosphodiester backbone or methylated base of the nucleic acid provide few opportunities for distinguishing between ribonucleotides and deoxyribonucleotide substrates, which is consistent with the ability of AlkB to function on both DNA and RNA.

**Figure 3:**
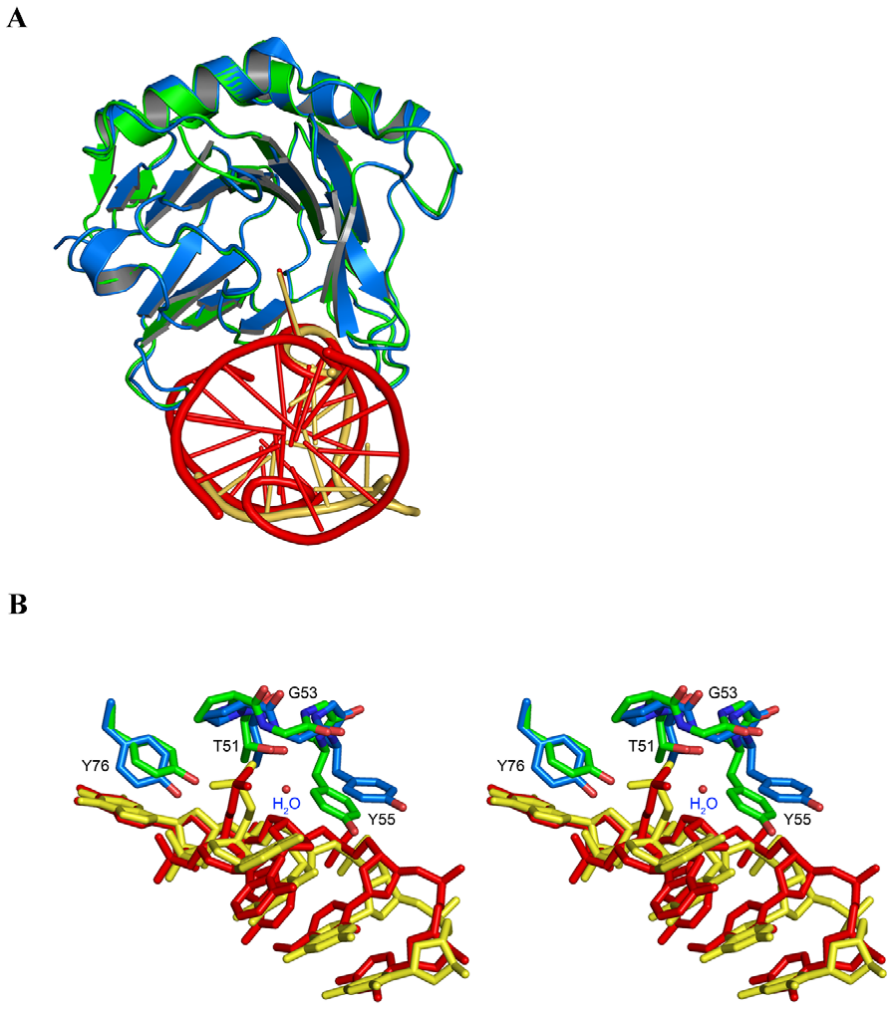
Structural comparison of AlkB:DNA complexes. (**A**) Least-squares superposition of D135A AlkB:ssDNA (green & yellow) with wt AlkB:dsDNA (blue & red) (pdbid: 3BIE) shows a very similar conformation of the protein in both structures with a r. m. s. d. = 0.912 Å and similar protein contacts with the damaged DNA strands. (**B**) Stereo image of the DNA binding loop and Y76 within the D135:ssDNA and wt:1-meA dsDNA complexes shows similar conformations of the nucleic acids and interactions with the DNA backbone by the T51-Y55 loop and Y76.

A least-squares superposition of our AlkB:ssDNA structure onto the previously determined AlkB:dsDNA structure (pdbid: 3BIE)[20] reveals the two complexes are very similar (core r.m.s.d. = 0.912 Å) (Figure 3). Binding of ss- and dsDNA to the AlkB protein appears to occur through equivalent interactions (Figure 3). The structural similarities of these two complexes further supports the idea that the lower efficiency of AlkB in repairing damaged bases in double-stranded DNA substrates is due largely to the higher energetic cost of base-flipping in from a DNA duplex.

### Damaged Base Searching and Binding

**Figure 4:**
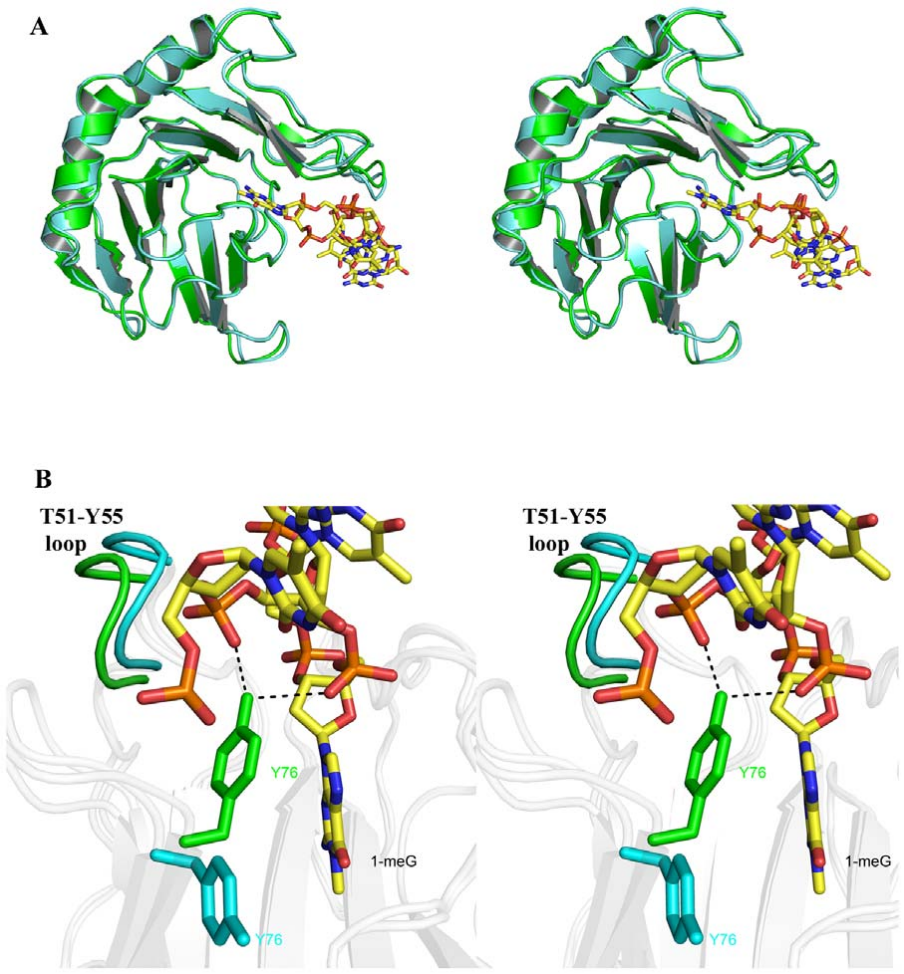
AlkB-DNA complexes. (**A**) (Stereo image) A superposition of the D135A AlkB:ssDNA (green) and unliganded wt AlkB (cyan) structures reveals very similar conformations in the overall structure of the enzyme between the bound and unbound states except in the active site. (**B**) In the presence of DNA, the T51-Y55 loop moves 2.5 Å to widen the binding groove and residue Y76 rotates from an ‘open’ conformation in the absence of DNA to close off the binding pocket when a methylated base is present. The rotation of Y76 in the presence of DNA also allows it to make hydrogen bonding interactions with the phosphodiester backbone.

The 90 amino acid N-terminal region of AlkB has been termed the “nucleotide recognition lid” subdomain, because backbone hydrogen/deuterium exchange studies have suggested that this domain is flexible in the absence of DNA [19]. Superposition of our structures of the AlkB-ssDNA complex with the unliganded AlkB proteins (core r.m.s.d = 0.772 Å) reveals little difference structurally in the global conformation of the proteins (Figure 4). Instead, our data indicate that AlkB is a fairly rigid molecule, and the flexibility and dynamics of the nucleotide recognition lid is more localized to residues Y76 and the T51-Y55 loop and not the whole domain, although we cannot rule out movement of this domain during intermediate DNA binding steps.

Greater insight into DNA binding and substrate base recognition comes from a comparison of the AlkB-ssDNA complex with the AlkB holoenzyme structure. In the absence of bound substrate, residue Y76 in the active site cleft adopts a conformation that is flipped away from the active site providing open access for an incoming base (Figure 4). The hydroxyl side chain of T51 is positioned to make hydrogen bonding interactions with the backbone amide nitrogen of residue Y55 to stabilize the conformation of the T51-Y55 loop. The presence of oligonucleotide in the structures of the ss- and dsDNA complexes has forced the widening of the binding groove through a shift of the T51-Y55 loop away from the active site by 2.5 Å. Additionally, with DNA bound and a substrate base in the active site, the Y76 side chain rotates to close the active site pocket. The result of this gating action seems to have several effects. In the closed conformation the side chain of Y76 provides a hydrophobic interaction with the substrate base and essentially clamps it into position for catalysis. Additionally, the hydroxyl group of the tyrosine side chain interacts with the phosphodiester backbone adjacent to the flipped nucleotide to further stabilize the catalytic complex. Previous analysis of the AlkB structure also suggested that the interaction of R161 with the phosphate of the methylated nucleotide was necessary for the recognition of this minimal substrate [19], [22]. Consistent with this observation, our structure of AlkB in complex with ssDNA containing 1-meG positions residue R161 nearby the phosphate oxygen of the substrate nucleotide flipped into the active site.

**Figure 5:**
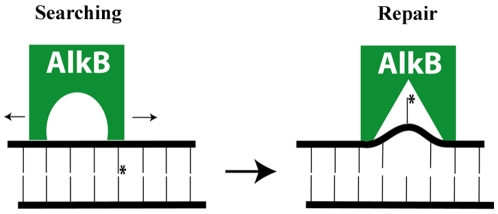
A model for ‘searching’ and ‘repair’ conformations of *E. coli* AlkB when bound to DNA. Site-directed mutagenesis studies coupled with DNA binding and activity assays reveal that binding damaged and undamaged DNA can be functionally separated suggesting there are at least two binding states for the AlkB protein as it searches for methylated bases.

In order to understand the roles of residues T51, Y76 and R161 in substrate recognition and DNA binding, we mutated each of these amino acids to alanine and compared the DNA binding of the mutant proteins on damaged and undamaged DNA as well as the rate of catalytic activity to wild type AlkB. Our results reveal the AlkB protein has at least two different DNA binding modes that we have termed the ‘searching’ and ‘repair’ conformations (Figure 5). Furthermore, these modes can be functionally separated by mutations that have greater effects on either ‘searching’ for damage or binding a damaged base in the active site for ‘repair’. DNA binding of the wild-type protein has about a 10-fold higher affinity for DNA containing an alkylated base compared to undamaged DNA (Table 2). The T51A mutation of the AlkB protein results in no change in affinity for damaged DNA (both ∼2 µM) but lowered the affinity for undamaged DNA from 16 µM to 51 µM creating the largest difference in binding affinities between damaged and undamaged DNA of any of the mutants we studied. This loss of affinity for the undamaged DNA suggests the T51-Y55 loop participates in the initial binding of the protein to DNA in the damage searching conformation of the protein. The fact that the T51A mutation has little effect on the binding of the protein to damaged DNA implies that once a methylated lesion located, other elements within the protein are able to effectively interact with the DNA and bind the damaged base. Interestingly, the rate of the T51A mutant in repairing a 1-meA lesion was also reduced to 41% of the wild-type activity. This reduction in activity is consistent with a decreased ability of the mutant AlkB protein to search for damaged bases and if so it would further imply that locating damaged bases in DNA is the rate limiting step.

**Table 2:** Rates of wild type and mutant AlkB enzymes on ssDNA containing 1-methyladenine and equilibrium binding constants on damaged and undamaged ssDNA.

	*Rate (µM/min)*	*Percent Wild Type (%)*	*1-meA (K_d_)*	*εA (K_d_)*	*Undamaged (K_d_)*
wt	0.32±0.05	100	2.0 µM±0.3	1.2 µM±0.1	16 µM±3.2
R161A	0.37±0.03	100	9.6 µM±0.7	8.0 µM±1.2	20 µM±1.3
W69A	N.D.	-	5.2 µM±0.7	7.8 µM±2.2	35 µM±6
D135A	N.D.	-	0.316 µM±0.03	0.127 µM±0.04	4.9 µM±1.3
Y76A	0.06±0.01	19	11 µM±1	22 µM±5	38 µM±1.6
T51A	0.13±0.02	41	1.5 µM±0.07	2.7 µM±0.5	51 µM±4

In contrast, the R161A mutation of the AlkB protein retains nearly wild-type affinity for undamaged DNA (20 µM) but shows close to a 5-fold decrease in affinity for damaged DNA (∼10 µM), indicating this residue has a larger role in the binding of the damaged base in the repair conformation than in the searching conformation (Table 2). Additionally, the rate of protein catalyzed repair of a 1-meA lesion using the R161A mutant is the same as wild-type protein. This is also consistent with the idea that lesion searching is the rate limiting step in the reaction and that once the lesion is located, oxidative repair is relatively fast. Residue R161 is positionally conserved in the structures of the human homologues, ABH2 and ABH3 [20], [21], suggesting they may use a similar mechanism in interactions with damaged nucleotides.

Mutation of residue Y76 to alanine (Y76A) shows a 2.2-fold decrease in affinity for undamaged DNA (35 µM), and a 5.5-fold decrease in affinity for damaged DNA (11 µM). The Y76A mutation also exhibits a rate of methylation repair of about 20% of wild-type protein. Our observations in the structures indicate that amino acid Y76 rotates to close the active site after binding a methylated base, which led us to predict that this residue plays a role in both damage base searching and catalysis. Removal of the tyrosine side chain would preclude the closing of the active site after a damaged base is bound. The reduction in affinity for damaged DNA along with the reduced catalytic activity in the Y76A mutant protein are consistent with the idea that the conformational change of this tyrosine from an open to closed configuration is important for both binding of damaged bases and catalysis. Furthermore, the reduction in binding undamaged DNA indicates that Y76 also plays a role in interrogating DNA for damage.

### AlkB Substrate Specificity

The structure of the AlkB protein in complex with ssDNA containing 1-meG shows the methylated base bound in the active site and stacked between W69 and H131 (Figure 2, 4). The O6 oxygen of the methylated guanine is pointed towards the space created by the absence of a carboxylate side chain in the D135A mutation supporting the idea that residue D135 provides selectivity for binding adenine and cytosine bases in the active site [11], [22]. A least-squares superposition of this structure with AlkB:dsDNA (pdbid: 3BIE) demonstrates that the 1-meG and 1-meA lesions are bound in very similar conformations (Figure 3) [19], [20]. Both lesions are flipped into the substrate binding pocket where the metal binding residues (H131, D133, and H187) and substrate binding residues (W69, mutant A135, wt D135) superimpose very well.

Mutation of residue D135 to Ala (D135A) in AlkB results in a ∼10-fold increase in affinity for DNA containing a methylated base and a 4-fold increase affinity for undamaged DNA. Most interestingly, the rate of repair for this mutant protein on a 1-meA lesion decreased to less than 5% of wild-type activity, but the repair of 1-meG lesions increased from undetectable levels to a rate of about 30% of wild-type activity on 1-meA (Table 2, 3). These data combined with the structure of 1meG bound to the D135A mutant lead us to propose multiple roles for residue D135 in substrate specificity and catalysis. First, the structure and activity assays support the idea that the carboxylate side chain of this residue interact with the exocyclic amines of methylated adenine and cytosine to provide selective binding of these bases in the active site. Removal of this residue not only relieves potential electrostatic clashes with guanine and thymine bases but provides additional space in the active site for tighter binding of even larger alkylated bases such as εA (Table 2). The fact that the D135A mutant protein displays an increased affinity for undamaged DNA to nearly that of wild-type protein with damaged DNA implies that this residue is involved in both the searching and repair steps of the AlkB mechanism and that undamaged bases are transiently sampled in the active site during searching of ssDNA. A second role for the D135 residue seems to be in facilitating catalysis. The rate of repair activity on 1-meA decreased by about 95% with the D153A mutant, even with an increase in affinity for a methylated base. Additionally, the repair of 1-meG with this mutant was 30% of wild-type activity in spite of the fact the structure shows the methylated base is correctly positioned in the active site for oxidative demethylation by the enzyme. It is possible that the D135 residue not only provides an interaction with the exocyclic amine of adenine and cytosine for substrate specificity, but this interaction contributes to catalysis, possibly through partial stabilization of the transition state.

**Table 3:** Rates of wild type and D135A enzymes repair of ssDNA containing 1-methylguanine.

	*Rate (µM/min)*
wt	0.005 µM/min±0.0006
D135A	0.22 µM/min±0.03

Residue D135 is strongly conserved in other AlkB homologues known to act on 3-meC and 1-meA. Interestingly, the human FTO protein has recently been identified as an AlkB homologue that acts upon 3-methylthymine and 3-methyluracil [17]. Consistent with our proposal that D135 plays a role in substrate specificity, the FTO protein contains an asparagine at this position which could provide a favorable interaction with the exocyclic oxygen of thymine or uracil and alleviate the potential electrostatic clash.

The AlkB protein containing the W69A mutation did not display any detectable levels of repair of methylated bases (Table 2). It also exhibited only a 2 to 3-fold reduction in DNA binding affinity for both damaged and undamaged ssDNA in comparison to the wild-type (Table 2). The complete loss of activity and relatively small loss in binding affinity in this mutant suggests that the primary role for residue W69 is to provide a π-stacking interaction for proper positioning of the substrate in the active site for catalysis (Figure 4). This is consistent with the presence of phenylalanine and tyrosine residues in the equivalent positions of ABH2 and AHB3, respectively, which maintain the aromatic nature of this amino acid. The small change in binding affinity for both damaged and undamaged DNA further suggests that W69 is also involved in searching for damaged bases, and again, that AlkB interrogates each base in searching for alkylation damage in ssDNA.

### Conclusions

The *E. coli* AlkB protein utilizes an oxidative dealkylation mechanism of removal of DNA damage and has diverged in higher eukaryotic homologues to prefer ssDNA, dsDNA, or RNA as well as different alkyl lesions. Here we present the structures of the full length *E. coli* D135A AlkB in complex with ssDNA containing a 1-meG lesion and the unliganded wild-type AlkB holoenzyme. Our combined structural and biochemical data show amino acids T51, W69, Y76, and R161 contribute to ssDNA binding, and substrate specificity. Our comparison of nucleic acid bound and unliganded structures reveals that residue Y76 undergoes a conformational change upon DNA binding necessary for damaged bases to bind in the active site and subsequent catalysis. Additionally, through site directed mutagenesis we were also able to functionally separate AlkB binding to damaged and undamaged DNA, leading us to propose two distinct binding modes for the protein in searching and repairing methylated lesions. Finally we show that residue D135 of AlkB provides substrate specificity for adenine and cytosine through favorable interactions with the exocyclic amines of the bases. Mutation of D135 to alanine allows tighter binding to other types of DNA damage and increases the rate of repair on 1-meG. These experiments have provided new insight into the mechanism of locating DNA damage in ssDNA by the AlkB protein.

## Materials and Methods

### Molecular Cloning, Expression, and Purification of *E. coli* AlkB

The *E. coli alkb* gene was PCR amplified from genomic DNA and inserted into a modified pET-19b expression vector (Novagen) that contained sequence coding for the Rhinovirus 3C protease recognition sequence (PreScission Protease, GE Healthcare) to permit removal of the N-terminal poly-histidine tag. The pET-19b-AlkB vector was then transformed into C41(DE3) *E. coli* cells for expression. One liter of LB-broth (Luria-Bertani) supplemented with 50 µg/mL of ampicillin was inoculated with C41(DE3) pET-19b-AlkB cells, grown to an OD_600_ = 0.5, and induced with 30 µM FeCl_2_ and 500 µM IPTG (isopropyl β-D-1-thiogalactopyranoside) for 4 hours at 25°C. Harvested cells were resuspended in lysis buffer (50 mM Tris 7.5, 500 mM NaCl, 15% glycerol). Cells were lysed using an EmusiFlex C-5 cell homogenizer (Avestin) and pelleting cell debris at 30000 x *g*. The supernatant was then passed over a Ni-NTA (QIAGEN) column equilibrated with lysis buffer. The column was then washed with 400 mL of lysis buffer containing 50 mM imidazole. AlkB protein was eluted in lysis buffer containing 500 mM imidazole. Fractions containing AlkB were then pooled and treated with PreScission Protease (GE Healthcare) according to the manufacturer's protocol and dialyzed against 30 mM MES 6.5, 150 mM NaCl, 2 mM dithiothreitol (DTT), and 15% glycerol for 12 hours at 4°C. Cleaved AlkB protein was then passed over a Superdex-200 gel filtration column (GE Healthcare) equilibrated with dialysis buffer. Peak fractions containing AlkB were pooled and analyzed for purity by SDS-PAGE electrophoresis. Purified protein was concentrated to 100 µM using the molar extinction coefficient 32430 M^−1^ cm^−1^ at λ = 280 nm. Concentrated AlkB was aliquoted and flash frozen in liquid nitrogen and stored at −80°C.

### Cloning, Expression, and Purification of *E. coli* AlkB Mutants

Mutations in the AlkB gene (T51A, W69A, Y76A, D135A, and R161A) were created by PCR using appropriate mutant primers to generate two halves of the mutant gene followed by annealing and PCR amplification of the two products using the primers described previously for the wt AlkB construct. Mutant constructs were expressed and purified following the procedure outlined for the wild type enzyme.

### Crystallization of Asp135Ala AlkB:1-meG DNA Complex and Unliganded wt AlkB

The D135A AlkB protein was buffer exchanged into 20 mM Bis-Tris 6.0, 100 mM NaCl_2_, 100 mM CaCl_2_ and concentrated to 5–15 mg/mL. The cofactors CoCl_2_ and 2-oxoglutarate (2-OG) were added to a final concentration of 1–3 mM and 5–20 mM, respectively. The protein was then mixed at a 1∶1 ratio with a 17-mer ssDNA oligonucleotide containing a single 1-methylguanine lesion (1-meG) (5′-TGCATCCATAXTGCCTG-3′, where X is 1-meG) (ChemGene, Inc.). The complex was crystallized by the sitting drop vapor diffusion method at 15°C at a 1∶1 ratio with a reservoir solution containing 10–20% w/v PEG-4K, 100 mM MES 6.0, and 100 mM MgCl_2_. Wt unliganded protein was buffer exchanged into 20 mM Bis-Tris 6.0 and 100 mM NaCl and concentrated to 5–20 mg/mL. Cofactors were added to a final concentration indicated above. The protein crystallized as described above in 10–20% w/v PEG-8K, 100 mM MES 6.0, 100 mM NaCl, and 100 mM MgCl_2_. Crystals grew within 1–2 weeks and were soaked into a cryo-protectant solution containing the reservoir solution plus 20% ethylene glycol, and frozen in liquid nitrogen for data collection.

### Structure Determination and Refinement

D135A AlkB X-ray diffraction data were collected at 100 K on a Rigaku MicroMax-007 rotating Cu anode generator with a Saturn-92 CCD detector. Wt AlkB X-ray diffraction data were collected on beamline X29A at the National Synchrotron Light Source (NSLS), Brookhaven National Labs. All data were integrated and scaled using the d*TREK program suite [24]. All structures were phased by molecular replacement using Phaser [25] with the AlkB structure (pdbid: 2FD8) as the search model. The models were built using the program *Coot* [26], and refinement was carried out using the programs CNS [27], and REFMAC5 [28], [29] with TLS refinement [30], [31]. All data collection and refinement statistics are found in Table 1. All molecular graphics figures were prepared using PyMOL [32]. Coordinates for the structures of the unliganded AlkB (pdbid: 3KHB) and the AlkB-ssDNA complex containing 1-meG (pdbid: 3KHC) have been deposited in the Protein Data Bank (PDB).

### Activity of AlkB Wild Type and Mutant Enzymes

AlkB catalytic activity was assayed using a 15-mer oligonucleotide containing a single 1-methyladenine (1-meA) base. Reactions contained 20 mM Tris 8.0, 6 mM Fe(NH_4_)_2_(SO_4_)_2_, 1 mM 2-OG, 10 mM ascorbate, 10 µM DNA (ChemGene) substrate, and 500 nM AlkB wild type or mutant enzymes. Reactions (10 µl) were carried out at 37°C for 10 minutes and quenched with 50 mM EDTA at 2.5 minute time points. Samples were prepared for matrix assisted laser desorption ionization time of flight mass spectrometry (MALDI-TOF MS) analysis using a C^18^ Zip-tip (Millipore) according to the manufacturer's protocols. Samples were mixed at a 1∶1 ratio with a 3-hydroxypicolinic acid matrix (10∶1 ratio of 3-hydroxypicolinic acid 50 mg/ml to 0.1 M diammonium hydrogen citrate) and spotted on a Bruker Daltonics MALDI-TOF MS MTP target frame III. Samples were analyzed in the negative ion mode on a Bruker Daltonics instrument. Peaks corresponding to the damaged substrate (4527 Da) and undamaged substrate (4512 Da) for the 1-methyladenine oligonucleotide were integrated using the area below the curve algorithm in SigmaPlot to determine the percent of the substrate repaired. 1-methylguanine (1-meG) activity assays were carried out under the same conditions outlined above with a 17-nucleotide 1-meG (ChemGene). Protein concentration was increased to 1 µM and reactions were carried out for 25 minutes. Peaks corresponding to the damaged substrate (5176 Da) and undamaged substrate (5162 Da) were integrated in the same manner as the 1-meA experiments. Percent of repaired substrate was converted to µM by multiplying by the total substrate in the reaction and plotted as µM vs. time to obtain a rate in the form µM/min. All experiments were carried out in triplicate and the data averaged.

### Fluorescence Anisotropy for DNA-Binding Measurements to Normal and Damaged DNA

Equilibrium DNA binding reactions for wild type and mutant AlkB proteins contained 45 mM Tris pH 8.0, 1 mM CoCl_2_, 1 mM 2-oxoglutarate (2-OG), and 1 nM 30-mer 5′- 6-carboxyfluorescein (6-FAM) labeled DNA oligonucleotide (Operon) containing either a 1-methyladenine lesion (1-meA), 1, N6-ethenoadenine (εA), or undamaged with increasing concentrations of AlkB wt and mutant enzymes. DNA concentrations were kept at 1 nM (≪K_d_) to insure equilibrium binding constants were being measured. CoCl_2_ was included to inhibit the enzyme from repairing the alkylated lesion as shown previously [13], [22]. Anisotropy measurements were carried out at 25°C (25 µl) in a microtiter plate on a Safire^2^ microplate reader with a fluorescence polarization module (Tecan). Polarization measurements were obtained using an excitation wavelength of 470 nm and emission wavelength of 525 nm. Data was normalized using the equation:
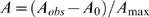
where *A_obs_* is the measured anisotropy at a given concentration of enzyme, A_o_ equals the anisotropy of DNA in the absence of protein, and A_max_ equals the maximum anisotropy observed. Data was fit to the one state binding model as follows:
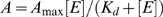
Results are the averages of triplicate experiments.
